# Determination of cut-off points for the Move4 accelerometer in children aged 8–13 years

**DOI:** 10.1186/s13102-023-00775-4

**Published:** 2023-11-28

**Authors:** Franziska Beck, Isabel Marzi, Alina Eisenreich, Selina Seemüller, Clara Tristram, Anne K. Reimers

**Affiliations:** 1 Department of Sport Science and Sport, Friedrich-Alexander-Universität Erlangen-Nürnberg, Gebbertstraße 123b, 91058 Erlangen, Germany; 2 Movisens GmbH, Augartenstraße 1, 76137 Karlsruhe, Germany

**Keywords:** Children, Acceleration, Measurement, Activity, Sensor

## Abstract

**Background:**

To assess physical activity (PA) there is a need of objective, valid and reliable measurement methods like accelerometers. Before these devices can be used for research, they need to be calibrated and validated for specific age groups as the locomotion differs between children and adults, for instance. Therefore, the aim of the present study was the calibration and validation of the Move4 accelerometer for children aged 8–13 years.

**Methods:**

53 normal weighted children (52% boys, 48%girls) aged 8–13 years (mean age = 10.69 ± 1.46, mean BMI = 17.93 kg/m^− 2^, 60th percentile), wore the Move4 sensor at four different body positions (thigh, hip, wrist and the Move4ecg including heart rate measurement at the chest). They completed nine activities that considered the four activity levels (sedentary behavior (SB), light PA (LPA), moderate PA (MPA) and vigorous PA (VPA)) within a test-retest design. Intensity values were determined using the mean amplitude deviation (MAD) as well as the movement acceleration intensity (MAI) metrics. Determination of activities and energy expenditure was validated using heart rate. After that, cut-off points were determined in Matlab by using the Classification and Regression Trees (CART) method. The agreement for the cut-off points between T1 and T2 was analyzed.

**Results:**

MAD and MAI accelerometer values were lowest when children were lying on the floor and highest when running or doing jumping jacks. The mean correlation coefficient between acceleration values and heart rate was 0.595 (p = 0.01) for MAD metric and 0.611 (p = 0.01) for MAI metric, indicating strong correlations. Further, the MAD cut-off points for SB-LPA are 52.9 mg (hip), 62.4 mg (thigh), 86.4 mg (wrist) and 45.9 mg (chest), for LPA-MPA they are 173.3 mg (hip), 260.7 mg (thigh), 194.4 mg (wrist) and 155.7 mg (chest) and for MPA-VPA the cut-off points are 543.6 mg (hip), 674.5 mg (thigh), 623.4 mg (wrist) and 545.5 mg (chest). Test-retest comparison indicated good values (mean differences = 9.8%).

**Conclusion:**

This is the first study investigating cut-off points for children for four different sensor positions using raw accelerometer metrics (MAD/MAI). Sensitivity and specificity revealed good values for all positions. Nevertheless, depending on the sensor position, metric values differ according to the different involvement of the body in various activities. Thus, the sensor position should be carefully chosen depending on the research question of the study.

**Supplementary Information:**

The online version contains supplementary material available at 10.1186/s13102-023-00775-4.

## Background

The assessment of physical activity (PA) and sedentary behavior (SB) in children has gained more and more attention in recent years, as obesity and other health risks are increasingly occurring in childhood [1]. As regular PA is beneficial for the health status [2–6] as well as social and cognitive development in children [7], valid, reliable and feasible measurement options are essential for research examining activity behavior [8]. Activity monitoring typically is divided into two categories: subjective and objective measurement methods [9–13]. Valid subjective measures like surveys, activity diaries or interviews are inexpensive and therefore they are often used to assess PA behaviors in large groups of individuals [14]. Nevertheless, they often have low accuracy and depend on children’s or their parents’ recall bias [15–17].

In contrast, technical measurement methods, such as calorimetry, doubly labeled water, pedometers and accelerometry [9, 18, 19], allow for an objective and non-invasive assessment of PA, place less burden on study participants, and are of particular importance for younger children who show difficulties to recall or estimate their past PA, and whose intermittent activity patterns make proxy reports very difficult [20, 21].

Far less expensive and complicated than calorimetry and double labeled water are accelerometers and pedometers [8]. In addition, they eliminate recall bias and social desirability [22, 23]. These methods are commonly used for objective assessment of PA [14, 24]. Pedometers typically contain a mechanical sensor that, when moving up and down, records that movement as a step [24–27]. In contrast to pedometers, accelerometers can measure movement acceleration in multiple planes. This makes it possible to quantify the intensity, frequency and duration of an activity and not just the number of steps taken [13, 28–31]. Furthermore, accelerometers can be worn on different body positions such as hip, wrist, chest or thigh. The common use are hip worn accelerometers, that reveal accurate classification of activity type and correct classification of PA intensities [32, 33]. Nevertheless, there are advantages of wearing accelerometers on other body locations such as improved comfort [33]. In addition, measurement accuracy and compliance has consistently improved during the last years [33–35]. In particular, wrist accelerometers were more often used in recent studies as it has been shown to reach superior compliance in children compared to hip placement [36]. Additionally, studies with accelerometers worn on the wrist or thigh revealed that they detected more specific activities and yielded acceptably accurate assessment of energy expenditure and SB [33, 37, 38]. Due to its advantages, accelerometers are commonly used to quantify PA in adults, adolescents, and children.

For any given accelerometer, thresholds need to be developed using a calibration and validation study. So far, there exist various calibration studies concerning different age groups in children and adolescents like toddlers, elementary school children and adolescents by implementing various approaches [21, 22, 39, 40]. Nevertheless, current accelerometer studies used different accelerometers. Furthermore, these studies mostly used regression models to compute the energy expenditure from the captured raw data [41, 42]. However, to predict regression models, a reference method like indirect calorimetry is necessary. Nevertheless, this method is quite expensive as well as time-consuming. Further, calorimetry is a laboratory-study and is not useful in free living conditions and practicable for children [20, 43, 44].

The accelerometer Move4 (movisens GmbH) used in the present study has already been extensively validated and tested for adults in this way [45, 46]. Nevertheless, children’s PA patterns and the resulting energy expenditure differ from adults and thus, separate cut-off points for PA intensities are need. Further, two different metrics exist that give the best possible indication of the intensity of physical movements and determine cut-off points for them for classification into activity levels. Thus, the research question of the present study is the calibration of the Move4 accelerometer for children aged 8–13 years. Specifically, we defined the following three aims:

Objective 1: Validation of the selected activities for the activity levels by using heart rate.

Objective 2: Modelling and determination of cut-off points to distinguish different activity levels (SB, light PA (LPA), moderate PA (MPA), vigorous PA (VPA)).

- by using different metrics (see study description): mean amplitude deviation (MAD) [47] and movement acceleration intensity (MAI) [48].
- by differing between the four sensor positions for mapping individual movement activities representation of cut-off points per movement activity per position.

Objective 3: Determination of the test-retest agreement by using data from two survey time points to determine the test-retest reliability of the Move4 together with the respective metrics.

## Materials and methods

### Study design

The longitudinal study is part of the EMPADIC (**E**xamination of **m**ethods for recording **p**hysical **a**ctivity and its **d**eterminants **i**n **c**hildren aged 8–13 years) – project. The study was approved by the local Ethics Committee (Ref. No. 56_20B) and was in accordance with the 1964 Declaration of Helsinki. All participants and their legal guardians provided written informed consent for the study participation. Data collection took place from May to September 2022.

### Participants and procedure

For the present study, we included children aged 8–13 years. Recruitment took place in local sports clubs and schools in Bavaria (Germany). All participants were included if they were healthy and had no known diseases that restrict them from doing sport. In school, all children were excluded who are exempt from sports lessons due to health restrictions or who are unable to participate in physical education lessons. After written informed consent of the board of directors of the sports clubs and the school director, we contacted various divisions in the sports club as well as teachers and asked, whether they would like to participate in our study. Then, parents as well as children obtained an information document and both had to give written informed consent for study participation.

For data collection, we used a test-retest design, to assess the validity of the cut-off points and the agreement between cut-off points between T1 and T1. There was a timespan of one week between the two measurement points. Data collection was pseudonymous, so at the beginning each participant received a personal code consisting of a combination of letters and numbers. Furthermore, for each child, weight and height, which was measured by a stadiometer (seca 813 and seca 213, seca GmbH & Co. KG), as well as the age, were documented. The children’s fitness status was recorded via the question “How many hours of exercise do you do per week?“ (see Supplement 1).

For the calibration of the Move4 for children, participants performed nine different activities outdoors under the guidance of the trained research team like lying, sitting, standing, running with different pace, stairs climbing, catching, and throwing a ball as well as jumping jacks. These activities consider four intensity levels: SB, LPA, MPA, and VPA. Table 1 lists the performed activities and the expected MET values. Data collection took place in a 90-minute training session (60 min test, 30 min of preparation and follow-up). Before the start of the test, the children’s verbal consent was obtained again. The sensors were applied to the children by the research team. For the present study, the children wore accelerometers at the following body positions: thigh (Move4); hip (Move4); wrist/ non-dominant hand (Move4) and chest (combination with ECG) (EcgMove4). When all sensors were properly attached, a jump was made to synchronize the sensors at each body position before the activities started. Such a jump was repeated after the activities have been performed. Subsequently, the children were instructed by the research team to perform each activity for a duration of four minutes. Average speeds for walking and running activities were estimated using defined distance and stopwatch. Further, a member of the research team guided the children through all activities and set the pace for the children for walking/running activities. This made it easier for the participants to maintain the estimated speed. During the implementation, the research team observed the participants and checked the correct performance of the activities. Between the four minutes activity there was a three-minute break to allow for transition between activities. After each activity children were instructed to stand still for 30 s to enable a separation of the activities. After completion of the nine activities the sensors were removed from the participants. Even if the rest between the activities was three minutes, we assume that the heart rate does not reach the resting heart rate after moderate and high intensity activities. As this could artefact the validation of the activities, we randomized the order of the activities for each data collection timepoint/study group.

**Table 1:** Activity intensities, tasks and expected MET values

Activity intensity	Activity type (based on Evenson et al. [22] and Trost et al. [49])	Expected MET from Youth Compendium of Physical Activities [50]
Sedentary Behavior (1.0–1.5 MET)	Lying Lying supine on a floor mat with the arms at the sides. Instruction to minimize all physical movements	1.2
	Writing Sitting on a chair writing a text with the arms on the table	1.4
	Standing Standing with the possibility of small movements in the hip	1.7
Light Activity (1.5–2.9 MET)	Slow Walking (≈ 2 km/h)	2.6
Moderate Activity (3–6 MET)	Normal Walking (≈ 4 km/h)	3.5
	Throwing and Catching Throwing and catching a ball, with a distance of 3 m, about 15 throws per minute	4.1
	Stairs climbing Climbing stairs with 80 steps per minute	6.0
Vigorous Activity (> 6 MET)	Running (≈ 7 km/h) Alternative: running as fast as possible	7.4 8.5
	Jumping Jacks	7.1

### Measurement instrument

The movisens sensor Move4 is a wearable device for measuring PA and sleep. The sensor EcgMove4 additionally records a single channel ECG and can be used to derive heart rate and heart rate variability. Both sensors have a 3-axis accelerometer and a 3-axis gyroscope for accurate detection of movements and body postures.

The sensor data is stored on an internal flash memory and can later be analyzed using the movisens Data Analyzer software [51, 52]. This software provides various analysis methods and visualization options to interpret the data and gain insights into PA, sleep and heart rate variability. Output parameters such as activity classes, body position, steps, energy expenditure and metabolic equivalents can be calculated.

The sensors are suited for use in scientific studies and interactive ambulatory assessment. They are used in a variety of applications, including sports performance analysis and rehabilitation [53–56].

The device is compact and lightweight, allowing for convenient and continuous use, and can be connected to a smartphone or other device for data transfer and analysis, e.g. to trigger questionnaires based on sensor data.

### Data analysis

Descriptive statistics were calculated for study variables, mean (M) and standard deviations (SD) for continuous variables and frequency (%) for categorical variables. We conducted a test for normal distribution (Kolmogorov-Smirnov and Shapiro-Wilk) for all continuous variables. The data analysis of the accelerometer data was divided into two major steps: data preparation and data processing. During data preparation, Matlab version 2015b (TheMathWorks, Inc.) was first used to trim the sensor data to the measurements of each child. The sequence of activities was then read in so that the individual activities and the breaks in between could be marked in the sensor data. This reference annotation was used to assign the calculated PA values to the respective activity.

If there was an error in data acquisition for example an interruption of the activities during the measurement, for example because a child had to go to the toilet, a note was added accordingly. In line with the annotations, these sections were marked in the respective measurement and excluded from further processing. Relevant parameters were also added for further processing. These included gender, age, heart rate and the measurement time point in order to better compare the two time points. The data was processed using Matlab (TheMathWorks, Inc.) and the movisens software [51, 52]. Thereby, the PA metric values for the MAD [47] and MAI (based on Van Someren et al. [48]) metrics were determined using the movisens DataAnalyzer algorithms. For MAI, the acceleration signal was bandpass filtered (Butterworth 0.25-11 Hz, 4th order) to remove parts that are not caused by bodily movement. The three axes were fused by the Euclidean norm. The final signal output was the mean value of the output interval. Movisens uses an output interval of one minute [57]. In the literature, the MAD metric is also called Vector Magnitude Count (VMC) and makes use of the mean signal value to reduce the effect of the constant gravitational acceleration [58].

To confirm the validity of pattern-based classification of intensity, Pearson correlation coefficients were calculated between the heart rates and MAD/MAI acceleration values. In addition, Matlab was used to create boxplots representing the heart rates across activities.

Subsequently, these metric values were used to determine the cut-off points for the four different activity levels in Matlab by a decision tree using the Classification and Regression Trees (CART) method [59]. This method is based on the recursive partitioning technique and creates decision trees by recursively partitioning the data until a predefined stopping criterion (maximum depth) is reached. The goal of the CART method is to minimize the variance in the data while maximizing the information in the data.

We determined the cut-off points for the different intensity levels for each sensor position and for both measurement time points. Finally, we analyzed the sensitivity and specificity of our analysis by using one minute of each activity as a reference value.

## Results

### Socio-demographics

In the study participated 53 children (52% boys, 48%girls) aged 8–13 years (mean age = 10.69 ± 1.46). Overall, participants had a mean Body Mass Index (BMI) of 17.93 kg*m^− 2^ (SD = 2.89) ranging around the 60th percentile [60] and regularly engaged in sports for 61.26 (± 26.16) minutes per day indicating overall a good fitness-level of the children. However, as seen in the standard deviation, children varied in their amount of sports activity per day. Test of distribution of the variables indicated a normal distribution of height, all other variables (age, weight, weight status, and sports activity) were not normal distributed. Further details can be seen in Table 2.

**Table 2:** Sample characteristics

	Overall	Boys	Girls
N (%)	53	28 (52%)	25 (48%)
Age [years; M ± SD]	10.69 (1.46)	10.73 (1.46)	10.66 (1.45)
Weight [kg]	38.92 (10.56)	39.13 (10.57)	38.78 (10.61)
Height [cm]	146.2 (11.40)	146.5 (11.27)	145.7 (11.10)
Body Mass Index (BMI) [kg*m^− 2^] (Range)	17.93 (2.89) 14.0–26.4	17.85 (2.97) 14.0–26.4	18.04 (2.80) 14.3–24.7
BMI percentile [60]		57.9th	61.8th
Sports activity [min/day] (Range)	61.26 (26.16) 21.4–128.6	69.16 (32.79) 21.4–128.6	52.86 (11.97) 34.3–85.7

### Mean values of each activity

Table 3 shows the accelerometer values for each activity broken down for MAI and MAD metrics. Overall, MAI values were higher than MAD. Mean accelerometer values were lowest when children were lying on the floor and highest when running or doing jumping jacks with no differences between boys and girls. The accelerations in each metric increase from intensity level to intensity level with the highest values for VPA.

**Table 3:** MAD and MAI metrics accelerations (mean absolute deviation, mg) in the nine activities. The lower part of the table presents the cut-off points from SB to LPA (Cut 1), from LPA to MPA (Cut 2) and from MPA to VPA (Cut 3)

	Hip		Thigh		Chest		Wrist	
	**MAD**	**MAI**	**MAD**	**MAI**	**MAD**	**MAI**	**MAD**	**MAI**
Activity	***Mean (SD)***	***Mean (SD)***	***Mean (SD)***	***Mean (SD)***	***Mean (SD)***	***Mean (SD)***	***Mean (SD)***	***Mean (SD)***
Lying	8.4 (5.2)	18.5 (15.5)	28.7 (46.3)	75.2 (123.5)	4.6 (5.5)	14.2 (15.3)	12.1 (19.1)	41.9 (55.6)
Sitting	10.9 (8.3)	37.2 (20.4)	55.0 (14.5)	155 (57.9)	5.9 (7.3)	24.6 (16.5)	28.2 (18.9)	84.5 (56.1)
Standing	26.6 (70.1)	49.3 (89.3)	25.4 (18.6)	90.9 (64.1)	23.9 (73.6)	45 (106.6)	38.6 (83.6)	86 (133.7)
Slow Walking	106.5 (33.6)	184.8 (42.8)	136.0 (6.9)	299.1 (32.9)	121.5 (39.5)	216.4 (49.5)	160.8 (61.6)	300.8 (102.4)
Normal Walking	215.9 (46.5)	306.9 (53.7)	209.9 (16.6)	394.5 (39.6)	243.2 (43.5)	357.7 (51.6)	248.0 (62.9)	402.7 (109.7)
Throwing and Catching	171.5 (67.4)	332.8 (95.3)	734.6 (24.6)	1159.5 (22.1)	162.5 (73.1)	294.1 (101.3)	549.3 (128.4)	929.7 (179.6)
Stairs Climbing	329.0 (94.6)	431.9 (115.2)	480.8 (57.1)	763.5 (84.1)	334.3 (98.7)	460.5 (123.1)	389.1 (128.5)	590.6 (198.1)
Running	832.7 (107.3)	1045.1 (135.1)	720.4 (28.7)	1364.3 (79.4)	763.2 (110.5)	1032.1 (139.5)	892.6 (139.4)	1599 (271.3)
Jumping Jacks	922.0 (135.4)	1070.2 (110.3)	725.9 (63.7)	1236.8 (65.2)	881.9 (98.3)	1023.9 (110.3)	975.8 (143.3)	1753 (193.1)

### Criterion Validity

**Fig. 1:**
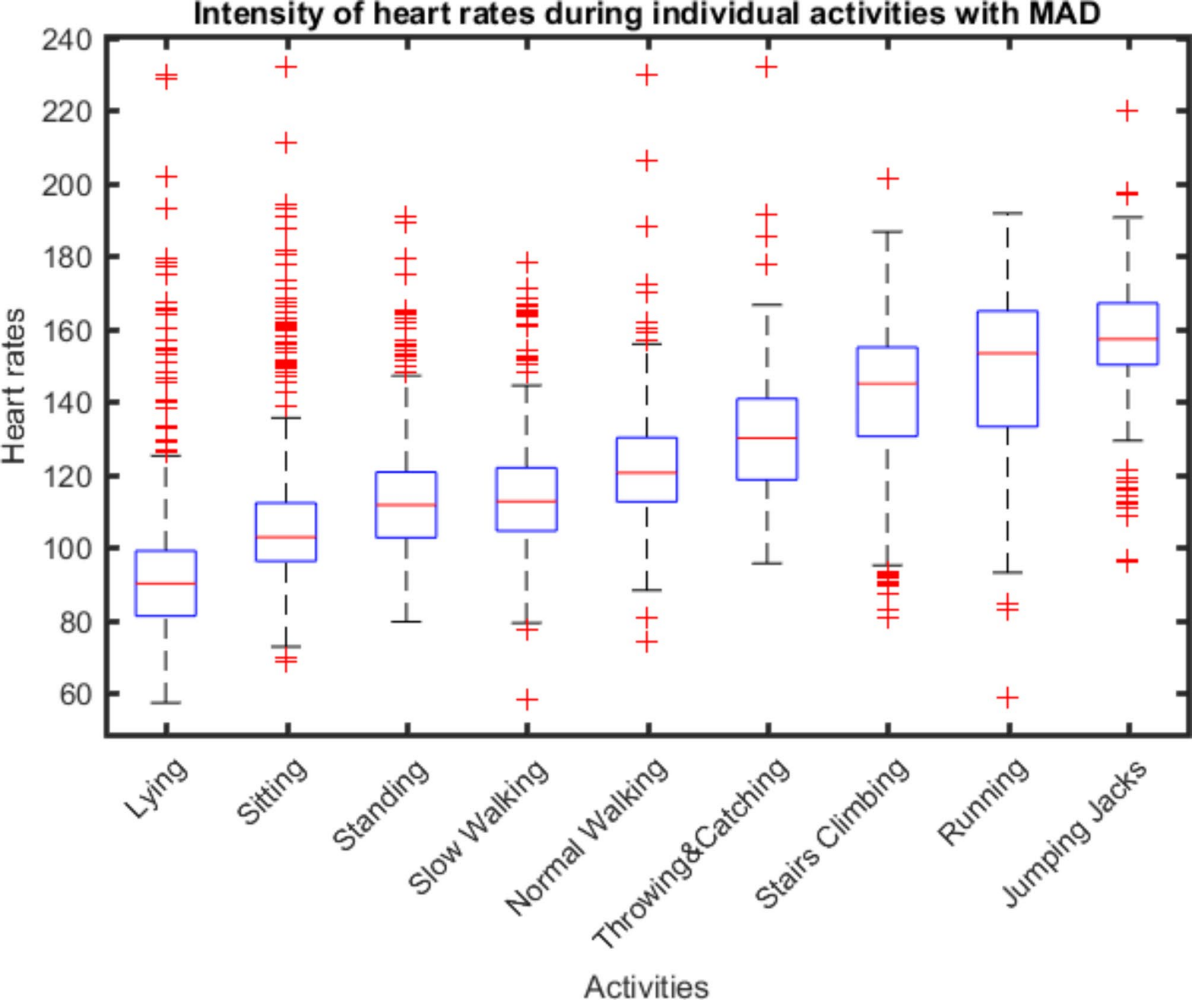
Intensity of heart rates during individual activities

Based on the individual mean values of each activity (MAD and MAI in mg), the criterion validity was then measured by correlation between the values of the MAD/MAI metric for each activity and the heart frequency (Ecg Move4). Overall, we had no limit of heart rate and thus no termination criteria. The mean heart rate for the SB activities was 105 beats (SD = 23) per minute (bpm), 115 bpm (SD = 18) for LPA, 132 bpm (SD = 20) for MPA and highest value for VPA with 152 bpm (SD = 21). The mean correlation coefficient between acceleration values and heart rate was 0.595 (p = 0.01) for MAD metric and 0.611 (p = 0.01) for MAI metric and thus, indicated a strong correlation between these the variables [61]. Figure 1 presents each activity with the associated heart rate.

### Cut-off points of intensity levels/thresholds

The nine activities considered four intensity levels (see Table 4) and validity showed a strong correlation between activities and heart rate and thus, cut-off points for SB-LPA, LPA-MPA as well as MPA-VPA could be determined. Table 4 shows the three cut-off points for the MAD and MAI metric for all four sensor positions. Overall, there were no meaningful differences in the cut-off points between boys and girls. Thus, we mention the overall cut-off points.

Overall, cut-off points differ between the two metrics with higher values for MAI across all sensor positions (see Table 4). In particular, the MAD values of the hip sensor are for SED (0-52.9 mg), LPA (53-173.3 mg), MPA (173.4-543.6 mg), VPA (> 543.6 mg).

**Table 4:** Overall cut-off points with MAD and MAI metrics (mg)

	Cut 1 (SB-LPA)		Cut 2 (LPA-MPA)		Cut 3 (MPA-VPA)	
Accelerometer	***MAD***	***MAI***	***MAD***	***MAI***	***MAD***	***MAI***
Hip	52.9	121.9	173.3	285.6	543.6	723.2
Thigh	62.4	182.5	260.7	457.7	674.5	858.3
Wrist	86.4	183.8	194.4	337.4	623.4	1058.9
Chest	45.9	101.5	155.7	240.2	545.5	672.8

### Sensitivity and specificity

Sensitivity and specificity for classification of each PA intensity category within the MAD metrics are displayed in Table 5. For SB, the wrist worn accelerometer had highest sensitivity (98.9%), but the hip worn has highest specificity (99.1%). For LPA, MPA and VPA, sensitivity (92.0%) as well as specificity (70.8%) were highest for the chest worn accelerometer. Overall, the chest position indicated the highest sensitivity (91.1%) and specificity (82.1%) values across all intensity levels for the MAD metrics.

**Table 5:** Sensitivity and Specificity of cut-off points (MAD metrics)

	SB		LPA		MPA		VPA		Overall	
Accelerometer	***Sens. (%)***	***Spec. (%)***	***Sens. (%)***	***Spec. (%)***	***Sens. (%)***	***Spec. (%)***	***Sens. (%)***	***Spec. (%)***	***Sens. (%)***	***Spec. (%)***
Hip	97.4	99.1	89.5	70.3	71.4	60.1	99.1	96.6	89.3	81.5
Thigh	98.3	98.9	87.8	66.9	44.8	59.5	91.9	99.0	80.7	81.1
Wrist	98.9	97.5	77.3	68.7	67.7	60.0	97.1	99.0	85.2	81.3
Chest	96.7	99.4	92.0	70.8	76.2	60.0	99.3	98.3	91.1	82.1

Sensitivity and specificity for classification of each PA intensity category within the MAI metrics are displayed in Table 6. For SB, the thigh worn accelerometer had highest sensitivity (98.2%), whereas specificity (99.7%) was highest for the chest worn accelerometer. For LPA, the thigh position indicated the highest sensitivity (93.9%) but the chest worn the highest specificity (73.6%). For MPA, sensitivity (76.8%) as well as specificity (60.1%) were highest for the chest worn accelerometer. Sensitivity (99.5%) for VPA was highest at the chest position and specificity (98.5%) at the wrist position. Overall, the hip position indicated the highest sensitivity (88.8%) and the chest position the highest specificity (81.7%) values across all intensity levels for the MAI metrics.

**Table 6:** Sensitivity and Specificity of cut-off points (MAI metrics)

	SB		LPA		MPA		VPA		Overall	
Accelerometer	***Sens. (%)***	***Spec. (%)***	***Sens. (%)***	***Spec. (%)***	***Sens. (%)***	***Spec. (%)***	***Sens. (%)***	***Spec. (%)***	***Sens. (%)***	***Spec. (%)***
Hip	95.8	99.5	91.4	70.7	69.0	60.1	99.2	94.4	88.8	81.2
Thigh	98.2	98.3	93.0	66.1	32.2	59.8	95.7	96.4	79.7	80.1
Wrist	97.9	97.9	65.3	68.4	61.2	47.3	75.8	98.5	75.1	78.0
Chest	81.6	99.7	89.6	73.6	76.8	60.1	99.5	93.4	86.9	81.7

### Test-retest agreement

As our study had a test-retest design, we compared the determined cut-off points for the intensity levels for all accelerometer positions between T1 and T2. Overall, for the MAD metric, the mean difference between T1 and T2 was 9.85% with greatest differences for the wrist worn accelerometer (16.3%) and lowest difference for the chest worn (6.4%). Independently of the accelerometer position, the highest deviation was seen for the SB-LPA cut and the LPA-MPA cut (see Table 7).

**Table 7:** Differences between T1 and T2 valued for the MAD and MAI metric (mg)

	Cut 1 (SED-LPA)			Cut 2 (LPA-MPA)			Cut 3 (MPA-VPA)			Overall
Accelerometer	**T1**	**T2**	**Difference**	**T1**	**T2**	**Difference**	**T1**	**T2**	**Difference**	**Difference**
**MAD METRIC**										
Hip	49.0	52.9	-8.0%	183.4	158.2	15.0%	549.1	542.0	1.0%	8.0%
Thigh	55.9	53.0	5.0%	283.7	247.0	14.0%	687.1	643.4	7.0%	8.7%
Chest	49.5	42.7	15.0%	157.6	155.4	1.0%	564.3	545.5	3.0%	6.4%
Wrist	94.4	76.3	21.0%	205.9	164.9	22.0%	623.4	664.5	-6.0%	16.3%
Overall			12.3%			13.0%			4.3%	
**MAI METRIC**										
Hip	130.5	115.1	13.0%	286.0	273.7	4.0%	760.5	723.2	5.0%	7.3%
Thigh	198.4	178.3	11.0%	484.5	457.2	6.0%	855.2	907.7	-6.0%	7.6%
Chest	100.3	109.5	-9.0%	237.8	223.3	6.0%	689.1	672.8	2.0%	5.6%
Wrist	180.4	211.5	-16.0%	337.4	385.1	-13.0%	1072.5	938.3	13.0%	14.0%
Overall			12.3%			7.3%			6.5%	

Similar results can be seen for the MAI metrics (see Table 7). Overall, the mean value of deviation between T1 and T2 was 8.63% with greatest differences for the wrist worn accelerometer (14.0%) and lowest difference for the chest worn (5.6%). Independently of the accelerometer position, the highest deviation was seen for the SB-LPA cut and the cut-off points for LPA-MPA and MPA-VPA had equal differences across all positions.

## Discussion

The aim of the present study was the calibration of the Move4 accelerometer for children aged 8–13 years. In more detail, firstly mean values for the selected activities (in mg for MAD and MAI metric) were assessed and then these activities and the energy expenditure were validated by using the heart rate. Secondly, cut-off points were modelled and determined to distinguish different intensity levels by using two different metrics (MAD and MAI) as well as by differentiating between the four sensor positions.

### Validity

First of all, the determination of activities and energy expenditure level was validated by using the heart rate measures. Overall, a strong correlation between the heart rate and the MAD values (in mg) of each activity was found. With increasing MET values, the heart rate of the activities increased. Based on this finding, it is possible to determine the cut-off points by using the MAD/MAI values of the activities. The present results are consistent with previous reports stating that heart rate is effective in detecting a variety of activity patterns [40]. Nevertheless, as we randomized the order of the activities, it was possible, that in some groups SB activities followed right after VPA activities. In that case, the three minutes rest could not have been enough time to normalize the heart rate. This could explain slightly higher heart rates of 105 bpm for SB compared to physiological studies indicating a resting heart rate of 90–95 bpm in this age range [62, 63]. In this context, it is worth mentioning that the validation of the activities was based on children with an overall good fitness level (mean activity minutes per day = 61.2) and a normal weight (overall BMI = 17.9 kg/m^− 2^, 60th percentile). Nevertheless, there were differences in the amount of activity and weight status between the children (see Table 2), resulting in differences in the HR while performing the activities [64]. This needs to be considered when interpreting the data. Still, as the mean value is quite good, we assume that the determined cut-off points can be used for children with a normal weight status and fitness level.

### Selection of activities

In this context, the selection of the activities for the present calibration study should be discussed. First of all, a combination of locomotor activities (e.g., slow walking with 2 km/h) and other free-play activities (e.g., throwing and catching a ball) was used to better simulate the different types of activities that children engage in. These were common to children of this age group and provided both, varying intensity levels and ranges of accelerometer counts [22]. This is also a principle suggested by Welk [65] and was used in several studies so far [66–69]. Furthermore, nine activities were used which is also applied in other studies [40, 41, 65, 68, 70]. The selection and classification of the used activities is based on the Youth Compendium of Physical Activity [50]. Unfortunately, only one activity (slow walking) was selected for LPA. Thus, it is suggested that further studies should apply an equal number of activities for each of the four PA intensity levels.

### Cut-off points

There is a need for using raw acceleration data instead of activity counts for measuring the intensity of PA [71–73]. Thus, the present study compared two different raw acceleration metrics (MAD and MAI) for the calibration of the Move4 sensor across four sensor positions in children aged 8–13 years. Within the discussion, further focus will set on the results of the MAD metrics, because, to the best of our knowledge, there is no validation and calibration study using the MAI metric so far. Nevertheless, this metric is also important as it uses a bandpass filter that ensures that accelerations that do not come from physical movements tend to be filtered out and is more and more used in studies [74, 75], so it was decided to determine the cut-off points for both MAD and MAI metrics. In comparison to Aittasalo et al. [70] who used the MAD metric for a hip worn accelerometer in children aged 13–15 years, our cut-off points differ especially in Cut 1 (SB-LPA: 52.9 vs. 26.9 mg;) and Cut 2 (LPA-MPA: 173.3 vs. 332 mg). One reason for the difference in the lower values in our study for LPA-MPA cut-off points might be the allocation of the activities to the intensities: In the present study, slow walking was the only activity for LPA and normal walking for MPA (oriented to the Youth Compendium of Physical Activity [50]), whereas Aittasalo et al. [70] allocated slow walking as well as normal walking to LPA. Another study using the MAD metric in 11 year old children indicated as the optimal cut-off points for LPA-MPA (= 3 MET) 91 mg and the MPA-VPA (= 6 MET) cut-off points was at 414 mg [47]. However, in this study participants performed a pace-conducted non-stop test on a 200 m long oval indoor track with initial speed of 0.6 m/s and it was increased by 0.4 m/s at every 2.5 min [47]. As free-living activities were also included, this could be the reason for the differences in the cut-off point from LPA to MPA (173.3 vs. 91 mg). In summary, our data show that the values respectively the cut-off points differ between the studies. This could be due to different samples, different activities and therefore it is important that for each sensor cut-off points are formed to make them usable for studies.

### Sensor positions

To the best of our knowledge, this was the first study comparing four sensor positions of any accelerometer. Existing studies compared in particular hip and wrist worn accelerometer [41] or hip, wrist and thigh [68], but none compared the hip, thigh, wrist and chest position for sensor location. As the different body positions are involved differently in the nine activities, it is not surprisingly that the cut-off points differ slightly across the four sensor positions.

Regarding sensitivity and specificity of each sensor position, our results indicated overall for the hip as well as for the thigh the highest specificity (MAD hip: 88.8%; MAD chest: 82.1%) as well as sensitivity (MAD hip: 89.3%; MAD chest: 91.1%) whereas the sensitivity and specificity for the wrist indicated in MAD metrics 85.2% respectively 81.3%. In contrast, Johansson et al. [41] indicated for wrist and hip worn sensors same sensitivity (SB: 100%, MVPA: 70%) and specificity (SB: 60%, MVPA: 100%) values in preschool children. Sensitivity and specificity values for two hip worn accelerometers indicated in children aged 13–15 years almost perfect values for all cut-off values (98.6-100%) [70]. The different values of sensitivity and specificity in various studies could be explained by the selection of the activities. Depending on how far the MET values of the activities are from the MET-cutpoint (e.g., 3 MET), there are different metric cut-off points and different accuracies in the detection. In comparison, a study investigating adults found high accuracy of the thigh-worn accelerometer for predicting time spent in each PA intensity category, as seen by sensitivities and specificities > 99% for correctly classifying each PA intensity category [68]. One possible explanation for the differences between children and adults could be the inconsistent performance of activities in children whereas adults could more consequently perform activities over a certain time period [76].

Furthermore, regarding the accelerometer output (in mg) within one intensity level, there are differences according to the body position to which the sensor is attached. In particular, the MAD metrics for SB varied widely in our study: 52.9 mg for the hip placement, 62.4 mg for thigh, 86.4 mg for the wrist sensor and 45.9 mg for the chest position. The high values of wrist worn accelerometers in SB could be explained by the fact that this sensor position captures movements performed by the arms, unlike a hip worn monitor [41]. Especially younger children have problems to stand still without moving their arms [41]. Our findings are in accordance with results from other studies [36, 41, 77] which showed higher values for wrist-worn accelerometer compared with hip worn sensors, while measuring simultaneously.

### Recommendations for sensor positions

We suggest to choose the sensor position depending on the research question. Overall, the sensor at the hip is really comfortable and shows good values and is already commonly used [32, 33, 78, 79]. Furthermore, the hip worn sensor indicated good sensitivity and specificity values in our study. Nevertheless other body positions should be considered while planning a study [68]. In particular, accelerometers worn on the thigh have shown high accuracy for measuring several different PA levels as well as SB and sleep [33, 38, 80–84]. Further, if there is an interest in the heart rate of the participants, the EcgMove4 accelerometer worn at the chest is suggested. Thus, it is easy to assess time spent in different intensity levels as well as the heart rate. The least favorable and efficient position seems to be the wrist due to low sensitivity and specificity.

### Test-retest agreement

Lastly, to assess the accuracy of the Move4 sensor, a test-retest design for the agreement of the cut-off points between T1 and T2 for all sensor positions was used. Overall, agreement indicated good values with small differences between T1 and T2. Regarding the cuts, Cut 1 and 2 indicated higher deviations compared to Cut 3. This could be explained by the types of activities within one PA level allowing greater variations in the execution. Especially during the SB activities (standing and lying), some children problems to hold the position and not to move their bodies, especially their arms. In contrast, VPA activities required the whole body to move which allows less variations in execution.

Regarding the sensor positions, only the wrist worn accelerometer showed great differences especially for SB-LPA and LPA-MPA cut-off points. A problem is that the wrist worn accelerometer output is highly depending on the movements of the hands [41]. In particular, the task standing for four minutes was highly challenging for some children and variances were recognized between the children (inter-individual) and also between the two measurement points (intra-individual) in relation to the movement of the hands. This could explain the differences of 16.3% between T1 and T2 cut-off points.

### Strengths and limitations

The main strength of our study is our sample size of 53 children aged 8–13 years which was numerous compared to other studies investigating between 20 and 47 participants [21, 41, 47, 70]. Furthermore, we calibrated and tested the Move4 sensor at four different body positions (hip, thigh, non-dominant wrist, and chest). Thus, the validation of the sensors was successful regarding a wide range of application possibilities. In addition, the different activities were not selected randomly, rather following the Youth Compendium of Physical Activity. Butte et al. [50] developed various activities and their resulting energy consumption in MET values. This is a meaningful list of activities and is a valuable resource. Furthermore, to ensure that the activities are performed with high accuracy, one research assistant was leading and participating in the exercise. This was highly important for the walking and running activities to lead the pace.

A limitation of this study relates to different weather conditions during the time period of the data collection. Therefore, some of the exercises were carried out indoors, which may have affected the children’s movement. Secondly, the sensors have partially fallen off during the movements. Although they were immediately reattached, a few seconds of activity had to be cut out. Further, some participants had difficulties to perform the activity the whole duration of four minutes. Thus, the data preparation contains cut outs to clean the raw data. Besides performing for four minutes, the accuracy of the execution lacked (e.g., standing). In this context, the validation of the activities and thus the determination of the cut-off points need to be slightly limited as the sample differed within the amount of activity (fitness level) and weight status, which might result in variability of the heart rate within one activity. Lastly, we only had one activity for the LPA intensity level that could be not representative for this level. Nevertheless, our data show good validity of the activities and the MET values. Further studies should consider that all intensity levels include more than one activity.

## Conclusion

This is the first calibration study using two different metrics (MAD/MAI) based on raw accelerometer data as well as determining cut-off points for four sensor positions using movisens Move4 sensor. Overall, our validation of the activities in regard to MET values by heart rate shows good correlation. Thus, the cut-off points showed good values for sensitivity and specificity. Test-retest agreement indicated good values with slightly more deviation from T1 to T2 in the wrist worn accelerometer. The optimal sensor position should be chosen depending on the research question of the study. Further calibration studies are needed for younger children, especially preschool children, as their activity patterns differ from children included in our study.

### Electronic supplementary material

Below is the link to the electronic supplementary material.

Supplementary Material 1

## Data Availability

The datasets generated during and/or analysed during the current study are available from the corresponding author on reasonable request.
