# Supplementary material for: Determination of cut-off points for the Move4 accelerometer in children aged 8–13 years
Source: BMC Sports Sci Med Rehabil. 2023 Nov 28;15:163. doi: 10.1186/s13102-023-00775-4 (PMC10683356; doi:10.1186/s13102-023-00775-4)
Supplement: Supplementary file 1 — Supplementary Material 1 [file 13102_2023_775_MOESM1_ESM.docx]

**ID: __ __ __ __ __ __ Date: __ __ __ __ __ __**

**Personal information**

Sex/gender: ð male ð female

Date of birth: _________________

Weight: _________________

Size: _________________

How many hours of sports do you do per week? _____________hours

**Accelerometer**

| **Position** | **Accelerometer number** |
| --- | --- |
| Thigh (right) |  |
| Hip (right) |  |
| Chest |  |
| Wrist (non-dominant) |  |

Start Time: ____________

End Time: ____________

Remarks:
